# Vital Statistics of the South-West in 1954

**Published:** 1956-04

**Authors:** A. H. Gale

**Affiliations:** Director of Medical Postgraduate Studies, University of Bristol


### VITAL STATISTICS OF THE SOUTH-WEST IN 1954

BY

A. H. GALE, D.M.

*Director of Medical Postgraduate Studies, University of Bristol*

The annual reports of the Medical Officers of Health of the five county boroughs and of the five administrative counties which are wholly or partly within the South-West Hospital Board Region, give much interesting information on the public health in the year 1954. The Statistical Review published by the Registrar-General (Part I) supplements the information in the annual reports.

Some of the fundamental vital statistics are set out in Table I.

TABLE I

PRINCIPAL VITAL STATISTICS

of County Boroughs and Counties in the South-West Region in 1954.

| County Boroughs | Population | Live Births | Deaths | *A* | *B* | *C* | *D* |
|---|---|---|---|---|---|---|---|
| Bath .. .. .. | 79,400 | 1,043 | 980 | 0·88 | 0·85 | 21 | 26 |
| Bristol .. .. .. | 444,900 | 6,703 | 5,165 | 0·98 | 0·99 | 21 | 23 |
| Exeter .. .. .. | 76,900 | 1,112 | 990 | 0·96 | 0·98 | 26 | 35 |
| Gloucester .. .. | 66,700 | 1,138 | 731 | 1·11 | 0·99 | 20 | 17 |
| Plymouth .. .. .. | 217,500 | 3,582 | 2,348 | 1·11 | 0·97 | 28 | 25 |
| *Counties:* | | | | | | | |
| Cornwall .. .. .. | 343,200 | 4,845 | 4,537 | 1·00 | 0·96 | 21 | 31 |
| Devon .. .. .. | 510,000 | 6,684 | 7,234 | 0·98 | 0·93 | 25 | 21 |
| Gloucestershire .. .. | 446,600 | 7,037 | 5,012 | 1·09 | 0·94 | 25 | 22 |
| Somerset .. .. .. | 485,000 | 6,987 | 5,901 | 1·02 | 0·90 | 22 | 20 |
| Wiltshire .. .. .. | 394,800 | 6,270 | 4,112 | 1·14 | 0·90 | 25 | 20 |
| *Whole Region:* .. .. | 3,065,000 | 45,401 | 37,016 | 1·03 | 0·93 | 24 | 23 |

*A.* Ratio of local adjusted birth-rate to national rate.

*B.* ,, ,, ,, ,, death ,, ,, ,, ,,

*C.* Infant mortality per 1,000 live births.

*D.* Stillbirth rate per 1,000 live and stillbirths.

### BIRTH-RATES

The birth-rates (col. *A*) of Gloucester C.B., Wiltshire and Plymouth C.B., were a little higher than the national rate and that of Bath was a little lower while those of the other areas were much the same. All the death-rates (col. *B*) were a little lower than the national rate. Infant mortality rates (col. *C*) for the county boroughs were not very different from those of the administrative counties, but the rather high rate for Plymouth is commented upon by Dr. Peirson. He draws attention to the fact that there were nine deaths from gastro-enteritis among children under two. Dr. Irvine comments on the high stillbirth rate in Exeter (col. *D*) the total number of stillbirths in Exeter was 41 but where numbers are as small as this rates will inevitably fluctuate from year to year.

DEATHS

Figures of deaths by age and cause for the whole region are given on pages 220–1 of the Registrar-General's Statistical Review, 1954 (Part I). They show how great have been the changes in the general picture of mortality in recent years.

The number of deaths at all ages in the year was 37,016, 18,713 of males and 18,303 of females.

*Infants under 4 weeks*

In this age group there were 740 deaths and not one was ascribed to the definite diseases classified as infective or parasitic. Only 70 were classified to one of the indefinite groups of infections. Nearly all the rest were returned under such headings as congenital malformations (122), birth injuries, &c. (285), immaturity and ill-defined diseases of early infancy (244).

*Infants 4 weeks to 1 year.* Total deaths 331.

Formerly the specific infectious diseases were important causes of death among older infants but in 1954 there were only 10 deaths from whooping-cough, none from measles and 5 from tuberculosis. Pneumonia (95), bronchitis (14) and gastroenteritis (25) were the most prominent of the infections. Congenital malformations accounted for 78 deaths and accidents for 26 (males 21, females 5).

*Pre-school children (age 1–4).* Total deaths 146.

The deaths from malignant disease (15) exceeded those from tuberculosis (9) and of the specific infectious diseases only meningococcal infection (2) caused any deaths at all. Pneumonia (18), influenza (3) and bronchitis (1) were credited with 22 deaths and accidents with 36 (males 23, females 13). Congenital malformations (16) were still important.

*School children (age 5–14).* Total deaths 210.

There were 30 deaths from malignant disease and 14 from all the definite diseases classified as infective and parasitic including 3 from tuberculosis. Influenza (2), pneumonia (16) and bronchitis (1) are not included under the comprehensive heading of "infective and parasitic". Accidents accounted for 55 deaths of which 23 were due to motor vehicle accidents. It is perhaps a reflection of the greater rashness of boys that 18 of those killed in motor accidents were boys and only 5 were girls.

*Adolescents and Young adults (age 15–24).* Total deaths 314 (male 238, female 76).

This used to be the age group in which there was a great excess of female over male deaths from pulmonary tuberculosis. In 1954 there were 9 deaths of females and 5 of males from this cause. There were 28 male and 7 female deaths from malignant disease, but the principal reason for the great excess of male over female deaths is to be found under the heading " accidents"—84 males and 9 females died as a result of motor vehicle accidents and 40 males and 1 female as a result of "all other accidents".

*Ages 25–44.* Total deaths, 1,360 (male 775, female 585)

In this group deaths from pulmonary tuberculosis were approximately the same in males (67) and females (61). Under the heading malignant disease there was an excess of female (169) over male (138) deaths. Deaths from vascular lesions of the central nervous system were much the same in the sexes—male 39, female 42—but deaths from arteriosclerotic heart disease were much higher among men (67) than among women (8). There were 28 male and 37 female deaths from chronic rheumatic heart disease. Once again accidents were a considerable cause of death—"motor vehicle", male, 56, female 5; "all other accidents", male 83, female 11.

*Ages* 45–64. Total deaths 7,708 (male 4,555, female 3,153).

Summary analysis is difficult in this age group because many causes contribute. The following are points of particular interest. There were 153 deaths of males from pulmonary tuberculosis and only 39 of females.

Deaths from malignant disease were 1,179 of males and 1,052 of females. The excess of male (486) over female (71) deaths from carcinoma of the trachea, bronchus and lung was largely offset by the female deaths from carcinoma of the breast (250) and uterus (131). Vascular lesions of the central nervous system caused 471 deaths of males and 526 of females whereas arteriosclerotic heart disease, &c. caused 907 deaths of males and only 259 of females. Under the somewhat indefinite headings of bronchitis and pneumonia there were 326 deaths of males and 120 of females.

*Ages* 65–74. Total deaths, 9,661 (male 5,255, female 4,406).

The main causes of death were similar to those in the preceding age group and the sex differences were much the same.

*Ages 75 and over*. Total deaths 16,546 (males 7,036, female 9,510).

In this age group the chief causes of death were degenerative diseases of the heart and blood vessels. Malignant disease was less prominent than in the preceding group.

The diseases which showed the most consistent changes in numbers of deaths in the five years 1950–54 were tuberculosis and carcinoma of the respiratory system. Deaths are shown in Table II.

TABLE II

DEATHS
(S.-W. Region)

| | | 1950 | 1951 | 1952 | 1953 | 1954 |
|---|---|---|---|---|---|---|
| Tuberculosis, Respiratory .. | | 923 | 776 | 594 | 506 | 445 |
| ,, Other .. .. | | 128 | 111 | 83 | 67 | 65 |
| Respiratory Carcinoma .. | M. | 557 | 615 | 656 | 710 | 835 |
| | F. | 110 | 130 | 144 | 132 | 162 |

INCIDENCE OF SICKNESS

Statistics of sickness are notoriously rudimentary in comparison with those of mortality, and the only comprehensive figures available are those of notifications of infectious diseases. Figures of notifications must always be treated with reserve but those for a succession of years do give some indication of trends. Tables III and IV give the figures for the more important diseases for the five years 1950–54. For convenience of comparison the diseases have been grouped in a different order from that of the Registrar-General's Review.

For some of these diseases it is possible to give figures of deaths but others do not appear in the abbreviated list of causes which is used for the classification of deaths by regions. In some instances, notably the indefinite group of respiratory diseases—influenza, pneumonia and bronchitis—the classification for notification purposes does not correspond with the classification for certificates of death. The figures of deaths show the epidemic element in the numbers of deaths from influenza, pneumonia and bronchitis. This is particularly obvious in 1951 and 1953 when there were epidemics of influenza due to virus A.

TABLE III

NOTIFICATIONS OF INFECTIOUS DISEASES
in the South-West Region, 1950–54.

| Disease | 1950 | 1951 | 1952 | 1953 | 1954 |
|---|---|---|---|---|---|
| Typhoid fever .. .. .. | 9 | 31 | 9 | 3 | 5 |
| Paratyphoid fevers .. .. | 19 | 34 | 27 | 13 | 14 |
| Dysentery .. .. .. | 489 | 1,420 | 783 | 474 | 1,152 |
| Food poisoning .. .. .. | 607 | 496 | 332 | 372 | 416 |
| Meningococcal infection .. | 52 | 51 | 76 | 75 | 52 |
| Poliomyelitis paralytic .. .. | 739 | 159 | 189 | 288 | 141 |
| ,, non-paralytic .. | 199 | 109 | 113 | 184 | 81 |
| Acute encephalitis infective .. | 24 | 63 | 8 | 8 | 11 |
| Acute encephalitis post-infectious .. .. .. | 3 | 9 | 6 | 8 | — |
| Scarlet fever .. .. .. | 3,816 | 2,949 | 3,566 | 2,966 | 2,264 |
| Erysipelas .. .. .. | 558 | 411 | 391 | 377 | 352 |
| Whooping-cough .. .. | 11,300 | 11,899 | 7,774 | 10,705 | 8,966 |
| Diphtheria .. .. .. | 56 | 55 | 29 | 35 | 9 |
| Measles .. .. .. | 14,703 | 50,829 | 13,361 | 52,034 | 6,355 |
| Acute pneumonia (Primary and influenzal) | 2,036 | 2,623 | 1,640 | 2,424 | 1,690 |

TABLE IV

DEATHS FROM NOTIFIABLE DISEASES
in the South-West Region 1950–54

| Disease | 1950 | 1951 | 1952 | 1953 | 1954 |
|---|---|---|---|---|---|
| Typhoid fever .. .. .. | | 3 | 1 | 0 | 1 |
| Dysentery .. .. .. | | 4 | 3 | 1 | 2 |
| Meningococcal infection . .. | 13 | 12 | 11 | 15 | 10 |
| Poliomyelitis .. .. .. | 104 | 24 | 24 | 32 | 10 |
| Scarlet fever and streptococcal sore throat .. .. .. | | 9 | 8 | 9 | 5 |
| Whooping-cough .. .. | 20 | 31 | 17 | 16 | 12 |
| Diphtheria .. .. .. | 2 | 2 | 4 | 2 | 0 |
| Measles .. .. .. .. | 8 | 18 | 4 | 16 | 0 |
| Influenza .. .. .. | 322 | 1,184 | 148 | 634 | 135 |
| Pheumonia .. .. .. | 1,195 | 1,568 | 1,191 | 1,467 | 1,272 |
| Bronchitis .. .. .. | 1,457 | 1,884 | 1,237 | 1,527 | 1,248 |

The most striking feature of the table is perhaps the low mortality from the specific infectious diseases excluding influenza. The place of influenza is difficult to define because of the difficulty of diagnosis but it seems that in a year when virus A is prevalent there may be an excess of something like 1,500 deaths over a year when it is not prevalent. Measles seems to have resumed the two year periodicity which was so obvious a feature of its behaviour before the last war.

Acute rheumatism is not notifiable throughout the region but it is notifiable in Bristol C.B. and Cornwall (Table V). Figures of deaths are for the whole region.

TABLE V

ACUTE RHEUMATISM: NOTIFICATIONS, DEATHS

| | 1950 | 1951 | 1952 | 1953 | 1954 |
|---|---|---|---|---|---|
| Notifications | | | | | |
| Bristol C.B.* .. .. .. | 26 (4) | 28 (7) | 37 (4) | 37 (4) | 33 (0) |
| Cornwall .. .. .. | 26 | 24 | 14 | 14 | 9 |
| Deaths .. .. .. .. | | | | | |
| Whole Region .. .. | | 29 | 25 | 20 | 11 |

* Deaths shown in brackets.

The figures are too small for conclusions about trends but they do show that acute rheumatism is a very different disease now from what it was twenty years ago (1935) when 116 new patients with rheumatic heart disease attended the cardio-rheumatic clinic in Bristol and a new block was being built at Winford for the treatment of such cases.

*General comment*

Everyone knows that infections have become less important as causes of death than they used to be and that malignant disease, degenerative diseases of the heart and blood vessels and accidents have increased in importance. It is, however, a little surprising to find how much the balance has altered, particularly in the younger age groups. The leading position of malignant disease and accident as causes of death in pre-school and school children are good examples of this change of balance. It so happens that poliomyelitis, the one infectious disease which has become more important of recent years, was not epidemic in the South-West in 1954 and so the total number of deaths (10) was comparatively small. Nine of these deaths occurred at ages over 15.

